# Assessing Nitrosamine Migration from Drinking Water Contact Materials Using a Validated LLE-GC-MS Method

**DOI:** 10.3390/molecules30112403

**Published:** 2025-05-30

**Authors:** Beatriz Antunes, Andreia Videira, Ana Penetra, Vitor V. Cardoso, Rui N. Carneiro, Cristina M. M. Almeida

**Affiliations:** 1 iMed.UL, Faculdade de Farmácia, Universidade de Lisboa, 1649-003 Lisboa, Portugal; beatriz-antunes@edu.ulisboa.pt; 2 Direção de Laboratórios, Empresa Portuguesa das Águas Livres, S.A.—EPAL, 800-003 Lisboa, Portugal; andreia.videira@adp.pt (A.V.); ana.penetra@adp.pt (A.P.); vitor.cardoso@adp.pt (V.V.C.); rui.carneiro@adp.pt (R.N.C.)

**Keywords:** GC-MS, LLE, materials, migration assays, nitrosamines, water

## Abstract

Nitrosamines (NAs) are toxic compounds associated with disinfection processes. Human exposure can occur through the hydraulic hoses and seals that are in contact with drinking water. This study develops and validates a chromatographic method to quantify 11 NAs in water leachates from four organic materials. The method is based on liquid–liquid extraction (LLE) followed by gas chromatography coupled with mass spectrometry (GC-MS). The method was validated by the application of several statistical tests, namely, linearity/working range, precision, trueness, and recovery tests. The GC-MS method showed a good linear range for all NAs with coefficients of determination (r^2^) higher than 0.9989, coefficients of variation of the method (CVm) lower than 2.5%, and PG < F (0.05; 1; N-3). The working range varies between 10 µg/L and 386.7 µg/L. The GC-MS method showed good precision under repeatability and reproducibility conditions with a relative standard deviation (RSD) lower than 12% and 10%, respectively. The GC-MS showed good trueness with a relative error lower than 20%. Matrix effects were significant, with recovery (Rec) values between 47% and 125% and an RSD lower than 20%. The limit of detection (LOD) and quantification (LOQ) ranged between 0.71 µg/L and 8.9 µg/L and between 2.3 µg/L and 29.8 µg/L, respectively. The method quantification limits (MQL) ranged from 0.0045 µg/L to 0.0378 µg/L. The sum of the MQL (0.2 µg/L) is lower than the reference limit of 0.3 µg/L for NAs in the leachates from the migration tests. Four organic materials were subjected to migration tests with demineralized and chlorinated water to assess their suitability for the water supply system. These materials met the NA specifications for use in the water network.

## 1. Introduction

Water intended for human consumption, usually called drinking water or tap water, is water free of pathogenic microorganisms and harmful substances [1]. In the water supply system, the water interacts with different materials, which can have the capacity to change its quality due to the leaching process. Therefore, water quality management in water supply systems must consider the exchanges between the water and the network materials [2].

The materials and chemicals used in water supply systems can interfere with water quality in different ways: (i) by altering the chemical characteristics of the water; (ii) by contributing to bacteriological development/microbiological activity; (iii) by changing the physical and organoleptic characteristics of the water; and (iv) by the migration of harmful substances [3,4].

The choice of material should be made considering the specifications for use, the characteristics of the water distributed (incrusting or aggressive), the location of the pipes, economic aspects, the fittings to be applied, and the heterogeneous mix of materials [4]. Depending on their formulation, materials can leach inorganic and organic substances into the water, which can affect the organoleptic properties of water or cause toxicological effects [5]. The nature of these interactions depends on the type of materials used. Materials can be grouped into three classes: (i) organic, which includes plastics, rubbers, silicones, coatings, and lubricants; (ii) metallic, which comprises metals, especially steel, copper, ductile iron, and metal alloys (e.g., brass); and (iii) cementitious, which are those that include cement in their composition (e.g., mortar, cement, and cement-based composite materials) [5].

Most of the materials in the supply system are organic, such as plastics, rubber, cement, enamel, and silicone, and can be used for sealants, reservoirs, pipes, coatings, and taps [6]. Organic materials represent a wide range of substances, including manufacturing by-products, polymerization aids, additives, dyes, pigments, and intermediate products that arise during the manufacture or decomposition by-products. These organic materials include accessories and their components, storage systems, and repair products. All these leached materials can react with the natural water compounds, namely, inorganic and organic compounds [7]. Included in the vast group of potential organic compounds from materials in contact with water are nitrosamines (NAs).

N-nitrosamines were first mentioned in the literature in 1853 as a product of the reaction of secondary amines with nitrous acid. However, it was not until 1956 that they began to gain notoriety [8]. The term NAs is used to refer to a large group of N-nitroso compounds (R_2_N-NO) of different molecular weights that contain a characteristic nitroso functional group (−NNO) covalently linked to their molecules [9]. N-nitrosamines are synthesized from nitrosation reactions because of the reaction of secondary and tertiary amines with nitrous acid or by the direct action of nitrosating agents, in which nitro groups are transferred from one molecule to another [10]. These organic compounds are volatile solids or non-hydroscopic liquids, depending on the structure of the precursor amine. They have a yellow or greenish yellow color due to the absorption of visible light by the NNO group [11]. The physical–chemical properties of NAs depend on their substituent groups. In general, low-molecular-weight NAs are liquid soluble in water, while high-molecular-weight NAs are only soluble in organic solvents. These compounds have boiling points between 150 °C and 220 °C and do not decompose when boiled. They are very stable compounds in neutral solutions and strongly alkaline solutions [12]. Their high stability in neutral solutions explains their occurrence and stability in the atmosphere, food products, and water. Under strong acidic conditions or ultraviolet (UV) radiation, NAs can decompose by cleaving the nitroso group [13].

These compounds are widespread in the aquatic environment and have at least four possible sources: (i) industrial or human contamination; (ii) microbial action; (iii) formation of disinfection by-products; or (iv) degradation of natural precursors [14,15].

In a water supply system, NAs can be formed by the reaction of disinfectant chemicals with secondary amines from contaminated water or materials used in the water network [16].

Extensive research has been conducted on NAs due to their potential carcinogenic, teratogenic, and mutagenic effects, even in low concentrations within the nanogram-per-liter (ng/L) range [15]. According to the available data, the Agency for Research on Cancer (IARC) classified most NAs as probably carcinogenic to humans (Group 2A) or possibly carcinogenic to humans (Group 2B). Only tobacco-related NAs are classified as carcinogenic to humans (Group 1) [17].

NAs have been identified in different types of water, such as wastewater, chlorinated drinking water, ozonated wastewater, and tap water (AT) [18,19,20,21]. The most detected nitrosamine in water is N-nitrosodimethylamine (NDMA), a by-product of water disinfection [15,22]. Seven nitrosamines, including N-nitrosodimethylamine (NDMA), N-nitrosomethylethylamine (NMEA), N-nitrosodiethylamine (NDEA), N-nitrosodi-n-propylamine (NDPA), N-nitrosopyrrolidine (NPYR), N-nitrosopiperidine (NPIP), and N-nitrosodi-n-butylamine (NDBA), have been widely detected in various waters and listed as target compounds in the method 521 of the U.S. Environmental Protection Agency (EPA) [23].

The World Health Organization (WHO) set a limit of 100 ng/L for NDMA in drinking water, which is associated with a 10^−5^ risk of developing cancer [24].

The US Environmental Protection Agency (EPA) established reference levels for other NAs: NDBA at 30 ng/L, NDEA at 0.4 ng/L, NDMA at 0.6 ng/L, NDPA at 7 ng/L, NMEA at 3 ng/L, and NPYR at 2 ng/L [23].

To quantify these NAs in trace concentrations, a sample pre-treatment is required to obtain analyte enrichment and matrix clean-up from undesirable interferers. A variety of approaches have been put forward for analyzing several matrices, including steam distillation [25], the celite column extraction method [26], liquid–liquid extraction (LLE) [27], pressurized liquid extraction (PLE) [28], solid-phase extraction (SPE) [29,30] and solid-phase microextraction (SPME) [31].

Common pretreatment methods for extracting and purifying NAs include SPE, LLE, and various microextraction methods, such as SPME and liquid-phase microextraction [32].

In this study, the LLE was selected to extract target NAs. The LLE is a technique widely used in the qualitative and quantitative analysis of organic compounds in water samples and is also one of the oldest sample preparation techniques. LLE provides huge linear sample capacity in comparison to other batch-type phase distribution techniques. This is so that there is less competition for space in the extract phase as its entire bulk-phase volume is available [33]. A second benefit is directly exposing the organic extract to a quantitative analytical measurement step, like liquid or gas chromatography. Desorbing the analyte from the extract phase is not required. Furthermore, “carryover” and other “memory” effects are minimal because each sample is extracted using a new quantity of solvent [34]. One last benefit of LLE comes from the extensive literature on solvent extraction that has been gathered over many years. This literature covers topics such as pH, organic solvent selection, and the types and concentrations of reagents (such as metal-chelation agents). It also discusses how these decisions impact selectivity, which is important for cleaning up samples, and quantitative extraction, which is important for preconcentrating the analyte component [34,35].

Quantification methods include liquid chromatography, gas chromatography, supercritical fluid chromatography, and electrochemical methods [30]. Several analytical methods based on liquid chromatography coupled with tandem mass spectrometry (LC-MS/MS) have been applied to quantify NAs in drinking water samples. However, LC-MS/MS shows relatively low sensitivity for some NAs [36]. On the other hand, gas chromatography (GC) has high sensitivity and good precision, but its disadvantage is the high matrix effect [37]. Currently, gas chromatography (GC) and mass spectrometry (MS) methods are used in combination. SPE is the best sample preparation technique for gas chromatography coupled with mass spectrometry (GC-MS), which allows for low detection limits for NAs in water samples [38]. GC-MS aims to increase the speed, sensitivity, and selectivity of methods adapted to water analyses [30,37].

Water for human consumption is in contact with different materials until it reaches the consumer’s tap. Due to the migration of some compounds and the oxidative process on the surface of materials, the water can change its organoleptic properties, which can alter the harmlessness of the water. Therefore, it is necessary to control the materials used in the supply system to guarantee the protection of human health [39]. The approval of materials to be applied in the supply system must comply with some requirements defined in a reference document relating to the material in question, which defines that tests have been carried out on the influence of the materials on the water for human consumption, following European or national standards. The European standards adopted for the assessment of manufacturing, non-metallic, and cementitious materials to be applied in the supply network regarding the leaching of organic materials are EN 12783-1 and EN 12873-2 [40,41].

Moreover, Directive (EU) 2020/2184 [1] on the quality of water intended for human consumption transposed into national law by Decree-Law 69/2023 [42] requires, among other things, an analysis of the potential risks associated with the building water distribution system and related products and materials, as well as the verification of the extent to which they affect the quality of the water at the point where it leaves the taps that are usually used for water intended for human consumption. Moreover, article 11 of Directive (EU) 2020/2184 requires that Member States ensure that the materials that come into contact with water intended for human consumption do not directly or indirectly compromise the protection of human health, adversely affect the color, odor, or taste of the water, enhance microbial growth, or leach contaminants into the water at levels that are higher than necessary given the intended purpose of the material [1].

Since 1998, the European Commission has been discussing how to implement a common approval scheme for materials in contact with water intended for human consumption. The 4MSI group (4 Member States Initiative) is responsible for developing a European approval scheme. Its sub-group developed several proposals, which were subsequently submitted to the European Commission and taken as a reference for a European approval scheme in Directive 2020/2184 [1]. The document written by this group, called “Requirements and test methods for products made of organic materials in contact with drinking water—4MSI Draft Common Approach on Organic Materials—Part C”, has the main objective of developing data for the construction of the European approval scheme for materials in contact with water and describes the maximum tolerable limits of compounds that can be found after the migration testing of materials used in drinking water distribution systems [43]. In this document, the guideline value or the maximum tolerable concentration at the tap (MTC_Tap_) for the sum of NAs is 0.3 µg/L [43].

To our knowledge, few studies have focused on this field. Therefore, evaluating the organic materials in contact with water for human consumption is mandatory.

This study aims to evaluate the potential contamination of drinking water with NAs from different organic materials used in water supply systems. To this end, this paper will (a) develop and validate an LLE-GC-MS method to quantify 11 NAs in drinking water; (b) select four organic materials that are commonly used in distribution networks, then carry out a series of migration assays on them using demineralized and chlorinated water for one month; (c) discuss the leaching of target NAs according to their potential sources and concentration limits in migration waters; and (d) assess the suitability of the materials for use in the water distribution network, considering their technical specifications and the specific requirements of the applicable standards.

This study addressed 11 NAs, namely, N-Nitrosodimethylamine (NDMA), N-Nitrosomethylethylamine (NMEA), N-Nitrosodiethylamine (NDEA), N-Nitrosodiisopropylamine (NDIPA), N-Methyl-N-nitrosobenzenamine (MNBA), N-Nitrosopyrrolidine (NPYR), N-Nitrosomorpholine (NMOR), N-Nitrosodipropylamine (NDPA), N-Nitrosopiperidine (NPIP), N-Ethyl-N-nitrosobenzeamine (ENBA), and N-Nitrosodibutylamine (NDBA).

## 2. Results and Discussion

A comparison of the results obtained in this study with those reported in the literature was difficult, primarily because LLE was used as the sample pre-treatment method.

The difference in results between LLE and more recent environmentally friendly approaches, such as SPE, is significant. This influences parameters such as the method quantification limit, recovery, and precision of the chromatographic method, making an objective comparison of analytical data impossible.

Concerning analytical calibration, although the chromatographic methods may differ, their performance can be analyzed via a comparison of their linearity, instrumental analytical thresholds, and chromatographic precision. For this reason, the results obtained were compared with those in the literature wherever possible and justifiable.

### 2.1. Chromatographic Run

**Figure 1:**
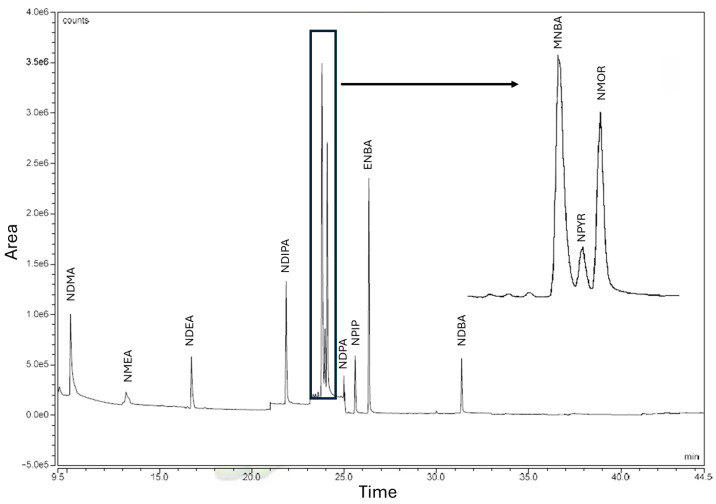
Chromatogram of a standard control with the targeted NAs: N-Nitrosodimethylamine (NDMA), N-Nitrosomethylethylamine (NMEA), N-Nitrosodiethylamine (NDEA), N-Nitrosodiisopropylamine (NDIPA), N-Methyl-N-nitrosobenzamine (MNBA), N-Nitrosopyrrolidine (NPYR), N-Nitrosomorpholine (NMOR), N-Nitrosodipropylamine (NDPA), N-Nitrosopiperidine (NPIP), N-Ethyl-N-nitrosobenzeamine (ENBA), N-Nitrosodibutylamine (NDBA).

The GC-MS method for NA quantification shows a chromatographic run of 35 min (Figure 1).

### 2.2. LLE Procedure

To achieve the guideline value of 0.3 µg/L for NAs in tap water (C_Tap_) [43], a concentration factor of 2000 is necessary. If the LLE is carried out with 500 mL of sample and for a final extract of 0.5 mL, the concentration factor is 1000, which is insufficient. However, with a sample volume of 1000 mL, the concentration factor is 2000, enabling us to meet the 4MSI requirement [43]. This accomplishment in the LLE optimization for NA analysis is a significant step forward.

**Figure 2:**
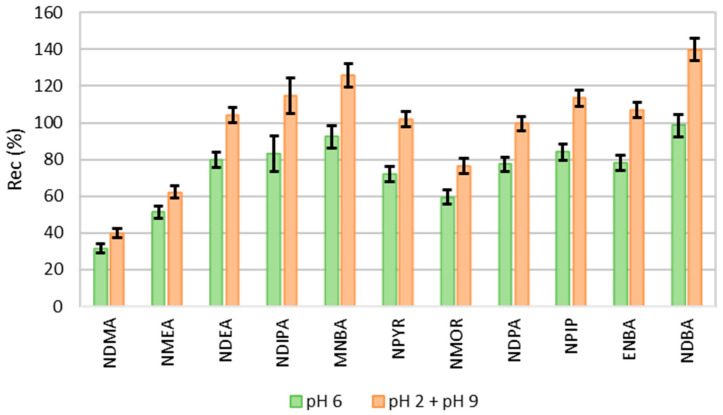
Recovery of NAs by LLE-GC-MS at pH 6 and both pH (pH 2 + pH 9) (n = 3).

pH is an important parameter in the extraction efficiency of analytes in aqueous matrices. In solvent–water systems, the partition coefficients of various solutes can change with the sample pH. It can change the ionization of the compound and, consequently, change its affinity for the organic solvent used in the extraction. Figure 2 shows the recovery of NAs when extracted at different pH values. The recovery rates are significantly higher (*p* < 0.05) when the sample is extracted in both acidic and basic conditions. The remaining tests were, therefore, carried out under these conditions.

Nitrosamines are chemical compounds with a secondary amine attached to the nitroso functional group. As a result, they behave differently depending on the pH, especially regarding protonation, solubility, reactivity, and stability, but they are generally stable in aqueous conditions [44].

Because the nitroso group lessens the basicity of the nitrogen, protonation at pH 2 is more difficult than with primary amines. Likewise, since no hydrogen atoms are bonded to nitrogen, deprotonation at pH 9 is irrelevant. Thus, the stability of the chemicals is the only element influencing recovery at basic or acidic pH values [44,45].

Other considerations, such as how nitrosamines are partitioned between water and dichloromethane, must be performed due to the intermediate polarity of these molecules. These compounds are substantially solvated (by hydrogen bonds) at a neutral pH, reducing their affinity to the organic solvent. Therefore, solvation decreases at basic and acidic pH values, which increases these chemicals’ migration to the organic solvent [44,46].

**Figure 3:**
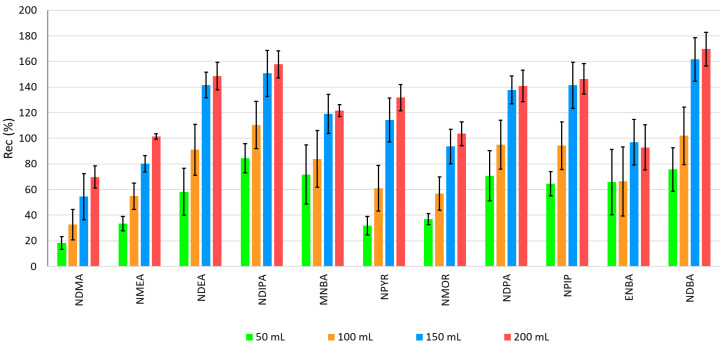
Recovery of NAs by LLE-GC-MS with different volumes of dichloromethane.

Regarding the volume of extraction solvent (Figure 3), the recoveries ranged between 18% (NDMA) and 84% (NDIPA) with 50 mL, 33% (NDMA) and 110% (NDIPA) with 100 mL, 55% (NDMA) and 162% (NDBA) with 150 mL, and 70% (NDMA) and 170% (NDBA) with 200 mL.

A greater volume of dichloromethane increases the dissolving capacity of NAs (apolar compounds) in this organic solvent. This enables the system to reach partition equilibrium more easily, particularly because these compounds are in low concentrations.

In real matrices (migration waters), potential coextracted interferents are more diluted, which lowers matrix interferences and improves measurement accuracy.

The matrix interferences are not representative in NA extraction from demineralized water, with a relative standard deviation (RSD) lower than 20% for all volumes of dichloromethane tested. However, the precision was also higher for 200 mL of dichloromethane: RSD < 12% for most target compounds.

Recoveries are significantly higher (*p* < 0.05) when 200 mL of solvent is used in the extraction, which is the volume selected.

### 2.3. Linear/Working Range

The chromatographic linearity range was first studied via an analysis of 20 standard solutions of NAs at different and equitably distributed concentration levels (2 µg/L–338.8 µg/L, preliminary linear range). The whole concentration range showed r^2^ values varying between 0.9697 and 0.9994, and CVm between 3.1% and 29%. Most NAs failed the statistical requirements. In this way, the whole concentration range was narrowed.

The best values were obtained for the concentration range (10 µg/L–386.7 µg/L) with r^2^ ≥ 0.9989 and CVm < 2.5% (Table 1).

In addition, the requirements of the Mandel test were fulfilled. Therefore, this linear range represents the working range of the method [47].

The coefficients of determination (r^2^) of the calibration curve of Nas by GC-MS are comparable to those described in the literature. However, they are higher than the minimum values of 0.996 and 0.9976 reported by Fu et al. [48] and Chen et al. [49], respectively.

Except for NDIPA and NPIP, with lower r^2^ values, all the other NAs showed r^2^ like those obtained by Sieira et al. [21] and Pozzi et al. [50] for a similar linear range, r^2^ ≥ 0.9992 and r^2^ ≥ 0.999, respectively.

The LOD and LOQ of the GC-MS method based on calibration curves (Table 1) ranged between 0.7–8.9 µg/L and 2.3–29.8 µg/L, respectively.

The LOD and LOQ of the GC-MS method under repeatability conditions (Table 1) ranged between 0.03–0.16 µg/L and 0.11–0.53 µg/L, respectively.

For most of the NAs, the LOQ is lower than the lowest concentration level of the working range.

For NA quantification, the instrumental LOQ was set to the first point of the working range.

When the working range is well adjusted and has no more than two orders of magnitude, and when the method is accurate, the experimental LOQ values are much lower than the first point of the working range. However, these values are not realistic. The errors associated with these concentration values are very high. Therefore, the first point on the curve should represent the LOQ [47].

While the analytical limits for many NAs are higher than those reported by other authors, the methods used to quantify them differ substantially. Regardless of the working day, the obtained values can be verified in subsequent chromatographic analysis, as this study adopted a more practical approach. In contrast, most publications used the signal-to-noise ratio (S/N) to determine their analytical thresholds [48,49]. Although this method may produce lower threshold values, suggesting greater sensitivity, it is unsuitable for routine analysis as it disregards variability in the analytical process, and the representative standards of these concentrations are neither precise nor measurable.

### 2.4. Precision

The data on method precision under repeatability and intermediate precision conditions for NAs by GC-MS are shown in Table 2 and Table 3, respectively.

The precision under repeatability conditions is lower for the upper concentration range, with RSDr between 0.80% (NMEA) and 11% (NPYR and NDPA). The RSDr in the lower concentration range varied between 1.1% (NDIPA) and 5.3% (ENBA) (Table 2). Under repeatability conditions, the GC-MS method showed good precision since the RSDr values were less than 25%. These values are like those obtained by other authors [19,48,49,50,51].

The RSD_R_ of the GC-MS method for NAs ranged between 5.6% (NMOR) and 8.8% (ENBA) (Table 3). As the RSD_R_ values are less than 25% and similar to those obtained in repeatability conditions, it can be concluded that the time variable does not significantly impact the method’s accuracy.

### 2.5. Recovery and Method Quantification Limit

The recovery studies were performed with ultrapure and drinking water (Table 4). Based on recovery results, the LOQs of NAs by the LLE-GC-MS method and the corresponding MQL were determined (Table 5).

The average recoveries of NAs in ultrapure water ranged between 57% (NDMA) and 106% (NDEA). For drinking water, the recoveries ranged between 47% (NDMA) and 125% (NDEA). Although the recovery profile is similar for both types of water, the recovery values in drinking water are mostly lower than those in ultrapure water, as observed by other authors [36,52,53,54]. For both waters, the RSD was lower than 20%.

The analysis of NAs in water mostly uses SPE, with recovery rates of over 80%. For example, McDonald et al. [51] obtained 81% and 111% recovery rates. However, most of these methods quantify a smaller number of NAs that do not always include our target compounds.

For example, Chen et al. [49], who analyzed nine NAS, showed recoveries ranging from 64% to 98%.

As the number of compounds increases, so does the difficulty of optimizing the most favorable extraction conditions given their different chemical structures and polarities. This work analyses 11 NAs. Therefore, the most favorable conditions for many of these molecules had to be selected, sometimes at the expense of others, to maximize the recovery of most of the target compounds.

Additionally, the lowest recovery values were found for NAs with a lower molecular weight and log K_ow_, such as NDMA and NMEA. These compounds are more soluble in water, resulting in a lower affinity for non-polar organic solvents [51].

Ideally, recoveries should be between 75% and 125%. However, when analyzing organic compounds in trace concentrations in complex matrices, recoveries tend to be lower due to matrix interferences. Moreover, the target NAs have low and similar molecular weights. Therefore, they have very simple mass spectra, whose molecular ion is of low intensity and similar to that of the potential interfering compounds in the sample. For these reasons, lower recovery values are acceptable with good precision. Therefore, recoveries lower than or equal to 50% were accepted since the RSD was lower than 25%.

The method quantification limits (MQL) ranged from 0.0045 µg/L (ENBA) to 0.0378 µg/L (NDBA). The MQL (0.2 µg/L) is lower than the guideline limit of 0.3 µg/L for NAs in the leachates from the migration tests [43]. Therefore, this MQL allows for the migration studies of target NAs from materials in contact with water and makes it possible to assess the suitability of various materials.

### 2.6. Nitrosamines in Migration Water

The migration tests were carried out on organic materials to assess their applicability to real samples (different materials). The parameters required for the calculations are summarized in Table 6. The results of the migration tests are shown in Table 7 and Table 8.

No NAs were detected in the migration waters of materials A and B.

The polyureas were produced by the quick interaction of isocyanates with polyamines. The migrating waters of these materials can show NAs whether nitrosating chemicals were used in their production or use. However, when properly formulated or manufactured, these materials are stable in normal use situations, reducing the possibility of dangerous compounds migrating [55]. The target nitrosamines were, therefore, absent from materials A and B (Table 7 and Table 8).

In the vulcanization process, rubber materials frequently employ secondary amine accelerators, such as thiurams and dithiocarbamates. These chemicals, used to cure rubber, are a known source of nitrosamine production [56].

Although polyols and isocyanates can be converted to polyurethanes, amine catalysts are frequently employed to regulate the process. Nitrosamines could also be produced if secondary amines are employed as catalysts and nitrosating agents are present (or develop during aging). Nitrosamine release risk may also be increased by degradation or insufficient cure [57]. Thus, it was not surprising that some NAs were present in migrating waters from materials C and D, especially from the rubber material (C).

Four nitrosamines were quantified in the migration waters of materials C and D, namely MNBA, NMOR, NDPA, and ENBA.

However, at the end of the third migration, only the migration water from material C (with and without chlorine) showed NAs above the LOQ. The migration water without chlorine showed 23 µg/L and 39 µg/L of MNBA and ENBA, respectively. The chlorinated migration water showed 50 µg/L and 58 µg/L of MNBA and ENBA, respectively. There was a moderate correlation between NA concentrations in the chlorinated and non-chlorinated migration water over the three migration tests, with a Pearson coefficient of 0.61. NA concentrations are around two-fold higher in the chlorinated migration waters.

Due to the oxidant property of chlorine, the rubber and polyurethane materials can be degraded. This degradation can open the polymer network, allowing entrapped or bonded NAs to migrate more freely into the water. However, chlorine may also react with potential residual amines or NA precursors in the rubber material, which can form new NAs under chlorinated conditions. Therefore, the NAs in chlorinated water can increase. Chlorination can also increase permeability and polymer swelling, facilitating the migration of small molecules like MBBA, NDPA, and ENBA to water. These NAs are relatively lipophilic and thermally stable, which makes them more likely to persist in the target materials and leach under chlorinated conditions [58].

In material C, the only one with NAs in the third migration, the C_Tap_ is 0.070 µg/L and 0.040 µg/L for migration waters with and without chlorine, respectively.

All the materials under study fulfil the requirements to be in contact with drinking water since the total concentration of NAs quantified in the consumer’s water (C_Tap_) is lower than the MTC_Tap_ (0.3 µg/L).

## 3. Materials and Methods

### 3.1. Materials

All nitrosamine standards are of analytical grade and with the highest purity available (purity between 90.30% and 99.90%), and were provided by Dr. Ehrenstorfer. Ultrapure water for in-house validation studies was obtained from an ultrapure water system, Mili Q (Millipore, Darmstadt, Germany). Dichloromethane for trace analysis (RS-Atrasol, 99.95%) and methanol for HPLC plus gradient grade (HPLC Plus, 99.9%) were provided by Carlo Erba. Individual stock solutions of 400 mg/L were prepared in methanol and stored at 5 °C ± 3 °C in the absence of light. The sulfuric acid and sodium hydroxide from Supelco were used for LLE. The migration studies were performed with demineralized water obtained by reverse osmosis, Elga Centra R200 (Veolia, High Wycombe, UK).

Free chlorine stock solutions were prepared by dilution from purchased 12% sodium hypochlorite solution (Sigma Aldrich, Saint Louis, MO, USA). Intermediate and standard solutions were prepared from sodium hypochlorite stock solution at concentrations between 0.5 and 10 mg/L.

### 3.2. Sample Selection

Four different organic materials (Table 9) used in the water supply system were selected and subjected to migration tests. The amount of material used for each test depended on each material’s surface-to-volume ratio (S/V).

The migration tests were performed over 11 days. They were carried out with chlorinated water (1 mg/L of chlorine) and demineralized water without chlorine. Demineralized water was also used as a test blank.

### 3.3. Liquid–Liquid Extraction

The target NAs were extracted using LLE. To optimize the LLE technique, various extraction conditions, namely the pH of the water samples and the volume of the organic solvent used for the extraction (dichloromethane), were tested.

The ultrapure water was spiked with 40 µL of NAs standard solution. Two different pH conditions were used to evaluate the recovery of the target compounds: (1) an extraction at pH 6 and (2) an extraction of same sample at both pH (2 and 9). The sample was acidified (pH 2) before extraction and then the sample was alkalinized (pH 9) before the last extraction. The effect of extraction volume was evaluated with four volumes of solvent extraction (50 mL, 100 mL, 150 mL, and 200 mL).

The extraction material (borosilicate glass-separating funnel and Turbovap tubes) was pre-rinsed with dichloromethane; 1000 mL of water samples were transferred to the glass-separating funnel and acidified with 6 mL of sulfuric acid (0.5 mol/L). A total of 50 mL of dichloromethane was added, and the water sample was shaken vigorously for 3 min. After the phase separation, the organic layer was collected into the Turbvap tube. One more extraction with 50 mL of dichloromethane was carried out in an acidic medium; 12 mL of sodium hydroxide solution (0.5 mol/L) was added to the aqueous phase, and the extraction with dichloromethane was repeated until 200 mL of organic extract was collected (two extraction in alkaline conditions). The organic extract was concentrated to 0.5 mL in the TurboVap evaporation system (Biotage, Uppsala, Sweden) at 25 °C and with a nitrogen (99.9995%) flow of 0.3 bar, resulting in a concentration factor of 2000. The final extracts were stored in 2 mL vials for chromatographic analysis.

### 3.4. GC-MS Analysis

The analyses were performed on a Trace 1300 gas chromatography coupled with an ISQ 700 mass spectrometer (Thermo Fisher Scientific, Monza, Italy) and electron ionization (EI) mode. Chromatographic separation was performed on a 60 m × 0.250 mm × 0.25 µm HP-5MS capillary column (Agilent, Palo Alto, CA, USA). Helium (99.9995%) was used as carrier gas with a constant flow rate of 1 mL/min. The injection was performed in splitless mode with a splitless time of 1 min. The injection volume was 1 µL, and the injector temperature was set at 250 °C. The transfer line and ion source temperatures were operated at 290 °C and 240 °C, respectively. The oven temperature ramp was programmed as follows: isotherm at 40 °C for 5 min, and then heated to 170 °C at a rate of 4 °C/min and increased by 20 °C/min up to the final temperature of 250 °C (3 min). The chromatographic run was performed in 44.5 min. Selected ion monitoring (SIM) mode was used for quantitation with a dwell time of 0.1 s (Table 10).

### 3.5. Quality Assurance and In-House Validation Studies

All the glassware was left overnight at 55 °C before use. For quality assurance purposes, at least one blank sample (BS), two standard controls (SCs), one duplicate (DD), and one recovery assay (REC) were performed for each batch of samples (daily analysis and chromatographic run), and they fulfilled the acceptance criteria defined in the laboratory for the analysis of trace organic compounds in water matrices (BS ≤ LOQ, standard error of SC ≤ 15%, DD ≤ 15%, and Rec = 100 ± 25%). The determination coefficients (r^2^) of calibration curves (daily calibration curves with a minimum of five standards) were higher than 0.995, and the coefficients of variation of the method (CVm) were lower than 5%. In this study, all NA concentrations lower than their detection limit were represented by not detected (n.d.).

The linear range was studied using an external calibration method at twenty concentration levels ranging from 2 µg/L to 338.8 µg/L. The linear range was evaluated by several statistical tests, namely the coefficient of determination (r^2^) and the coefficient of variation of the method (CVm, %). The working range was evaluated by the Mandel test [47,52].

The detection (LOD) and quantification (LOQ) limits of the GC-MS method were determined based on the standard deviation of the method (S_m_), namely LOD = 3 × S_m_ and LOQ = 10 × S_m_, respectively. S_m_ is the ratio between the relative standard deviation of the calibration curves (S_y/x_) and its slope (b). The LOD and LOQ were also determined under repeatability conditions. Ten replicate solutions with concentrations equal to the concentration of the first point of the linear range were analyzed by GC-MS. The standard deviations (SDs) were determined based on the areas for each compound. The LOD and LOQ were calculated using the formula (3 × SD) and (10 × SD), respectively [47,52].

The precision of the chromatographic method was evaluated under repeatability conditions and intermediate accuracy conditions. For repeatability studies, 30 standard solutions with NA concentrations corresponding to three concentration ranges were prepared: (i) 10 solutions with NA concentrations between 10 and 64.5 µg/L (first level of the working range), (ii) 10 solutions with NA concentrations between 20 and 128.9 µg/L (equivalent to the intermediate level of the working range), and (iii) 10 solutions with NA concentrations between 59.9 and 269.3 µg/L (equivalent to the higher level of the working range). The intermediate precision study was carried out over several days by analyzing a standard solution with NA concentrations between 20 and 128.9 µg/L.

The NA recoveries were also performed by spiking ultrapure water with 40 µL of a standard solution with NAs with concentrations between 20 and 128.9 µg/L. The analysis was performed with ten replicates. Due to the possibility of migration studies with tap water, the recovery was also performed with tap water under the same conditions.

The RSD (%) of the replicate analyses was determined for all target NAs and all fortification levels. The precision of the global method (LLE-GC-MS) was evaluated under repeatability and reproducibility conditions, and it was expressed as a relative standard deviation, RSD_r_ and RSD_R_, respectively. 

The individual LOQ of each NA and the method quantification limit (MQL) of the global method (LLE-GC-MS) for all NAs were calculated based on Equations (1) and (2), respectively.

(1) $$LOQ\left(x\right)=\frac{P1}{Rec\times FC}$$

(2) $$MQL=\sum_{1}^{11}LOQ(x)$$

LOQ(x) is the limit of quantification for each NA(x), expressed in µg/L, P1 is the concentration corresponding to the initial standard of the working range of that NA (µg/L), Rec is the average recovery of each NA (%), FC is the concentration factor, and MQL is the limit of quantification of the global method, LLE-GC-MS, for all target NAs (sum of NAs LOQ), expressed in µg/L.

### 3.6. Migration Assays

The migration tests were carried out with two types of migration water: chlorinated water (1 mg/L of chlorine) and non-chlorinated water. Demineralized water was also used for the blank assay, which was analyzed under the same conditions as the material migration tests.

**Figure 4:**
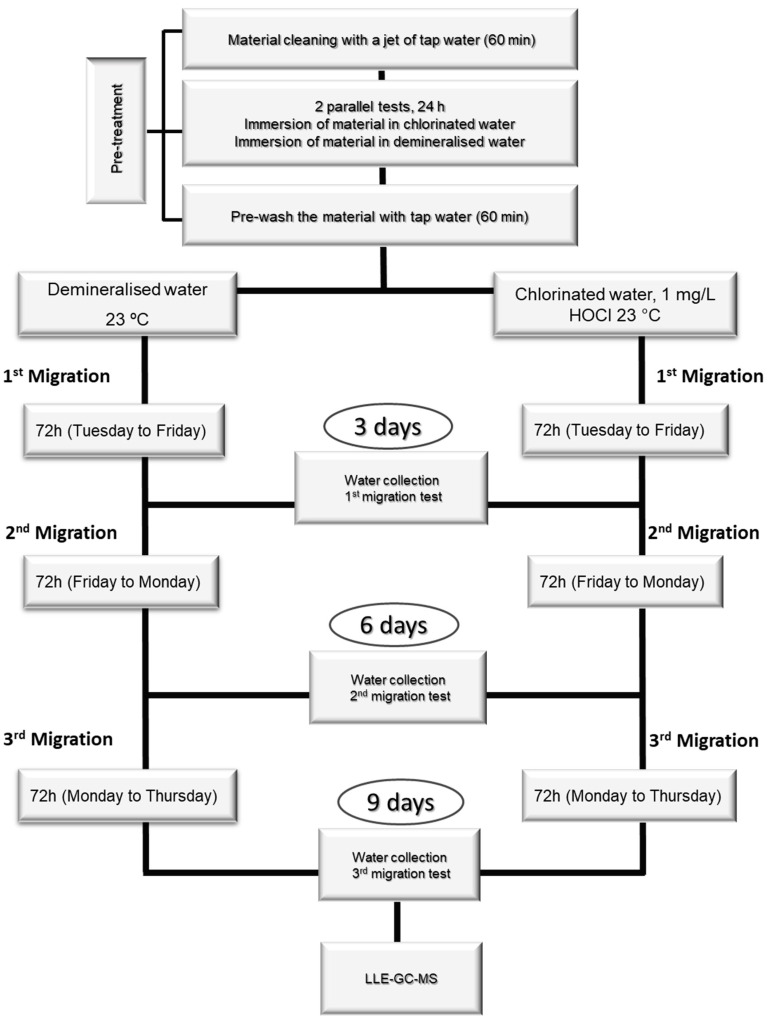
Material migration tests for NA monitorization.

The amount of material used for each test depends on each material’s surface-to-volume ratio (S/V). The tests were carried out over eleven days, with one day of pre-treatment, nine days of migration procedure, and one day of NA quantification by LLE-GC-MS (Figure 4).

The tests had a pre-treatment phase, which consisted of cleaning the materials with a jet of demineralized water for 60 min, followed by immersion in demineralized water for 24 h. After this treatment, the immersion water was discarded, and the materials were cleaned again with a jet of water for 60 min. After pre-treatment, the migration test consisted of immersing the materials in the test water for 72 h at 23 °C. At the end of these three days, the migration water was collected and analyzed by LLE-GC-MS. Two more migrations were carried out (3 migration assays, 9 days), and all the migration waters were analyzed.

The concentration of the NAs in the migration waters was obtained with Equation (3), where $c_{n}^{T}$ is the concentration of the substance leached from the material, expressed in mg/L (or µg/L), $a_{n}^{T}$ is the concentration of the substance in mg/L (or µg/L) measured in the migration water, and $b_{n}^{T}$ is the concentration of the substance in mg/L (or µg/L) measured in the blank test.

(3) $$c_{n}^{T}=a_{n}^{T}-b_{n}^{T}$$

The migration rate of a given substance ($M_{n}^{T}$—measured in mg dm^−2^ d^−1^ (or µg dm^−2^ d^−1^)) in the migration water is calculated from its concentration in that water, according to Equation (4), where t is the period of the migration test, expressed in days, 3 days in this test, and S/V is the surface area/volume ratio, expressed in dm^−1^.

(4) $$M_{n}^{T}=\frac{c_{n}^{T}}{t\times S/V}$$

According to Equation (5), the estimated concentration at the consumer tap (C_Tap_) was determined by considering the migration rate from the third migration test and the conversion factor (CF) of each material.

(5) $$C_{Tap}=M_{n}^{T}\times CF$$

## 4. Conclusions

The LLE combined with GC-MS provides a sensitive and accurate method for the trace analysis of 11 nitrosamines.

The GC-MS showed good linearity with coefficients of determination (r^2^) greater than 0.9989 (NDPA) and excellent coefficients of variation of the method (<2.5%). The LOD and LOQ of the GC-MS method under repeatability conditions ranged between 0.03 and 0.16 µg/L and 0.11 and 0.53 µg/L, respectively.

The precision of the GC-MS method is acceptable with RSDr values between 1.1% (NDIPA) and 5.3% (ENBA) for the first standard in the working range, and between 0.80% (NMEA) and 11% (NDPA and NPYR) for the highest concentration. Regarding intermediate precision, the RSD_R_ ranged between 5.6% (NMOR) and 8.8% (ENBA).

The average recoveries of NAs ranged from 47% (NDMA) to 125% (NDBA), with RSD lower than 25%. The LOQ of NAs by the LLE-GC-MS method ranged from 0.0045 µg/L (ENBA) to 0.0378 µg/L (NDBA), with an MQL of 0.20 µg/L. The LLE-GC-MS method showed good accuracy, and, therefore, it is suitable for analyzing NAs in materials in contact with water.

The migration water from materials A, B, and D did not show NAs (<LOQ). Four nitrosamines were quantified in the migration waters of material C with concentrations between 1.4 µg dm^−2^ d^−1^ (MNBA) and 18.6 µg dm^−2^ d^−1^ (ENBA). The C_Tap_ was 0.070 µg/L and 0.040 µg/L for migration waters with and without chlorine, respectively. Both values were lower than 0.3 µg/L.

All materials under study met the requirements to be used in the water network.

The types of organic materials used in distribution networks are not limited to the four materials under evaluation. Future work should, therefore, consider other organic materials.

Additionally, other potentially dangerous contaminants besides NAs may be leaching from the organic materials used in the distribution network, and these should be studied. Primary amines and other NAs should also be studied within the amine group.

The present study represents an important contribution to filling gaps in knowledge about the NAs in the leachates of materials in contact with water for human consumption. It is crucial for assessing potential health hazards. Therefore, it provides a step forward in ensuring compliance with material regulations and safeguarding public health.

Understanding the leaching capacity of certain materials enables water managers and distributors to assess their suitability for the water supply system more effectively, thereby minimizing exposure to harmful substances and safeguarding consumer health.

## Figures and Tables

**Table 1:** Working range, limit of detection (LOD), and limit of quantification (LOQ) of NAs in water samples by GC-MS.

NAs	N	Working Range (µg/L)	r^2^	CVm (%)	Mandel Test VT ≤ F (0.05; 1; N-3)	Calibration Curve		Repeatability	
						LOD (µg/L)	LOQ (µg/L)	LOD (µg/L)	LOQ (µg/L)
NDMA	8	24–109	0.9999	0.39	0.064 < 6.61	0.7	2.3	0.09	0.30
NMEA	9	30–180	0.9998	0.90	0.218 < 5.99	2.3	7.6	0.08	0.27
NDEA	9	60–359	0.9997	1.12	0.835 < 5.99	5.6	18.8	0.07	0.22
NDIPA	9	35–208	0.9995	1.43	0.098 < 5.99	4.2	13.9	0.03	0.11
MNBA	9	16–96	0.9998	0.91	1.43 < 5.99	1.2	4.1	0.08	0.27
NPYR	6	65–193	0.9992	1.34	7.17 < 10.13	4.9	16.2	0.08	0.26
NMOR	6	45–157	0.9994	1.34	5.78 < 10.13	3.8	12.7	0.06	0.19
NDPA	6	24–72	0.9986	1.74	4.82 < 10.13	2.4	7.8	0.06	0.21
NPIP	9	50–300	0.9989	2.12	3.29 < 5.99	8.9	29.8	0.09	0.29
ENBA	8	10–45	0.9997	0.94	2.58 < 6.61	0.7	2.3	0.16	0.53
NDBA	7	60–208	0.9992	1.38	4.38 < 7.71	5.2	17.3	0.11	0.35

**Table 2:** Precision under repeatability conditions for the first and last concentration levels of the working range of NAs by GC-MS.

NAs	Lower Concentration		Higher Concentration	
	C (µg/L)	RSD (%)	C (µg/L)	RSD (%)
NDMA	24.2	3.0	145	2.3
NMEA	30.0	2.7	180	0.80
NDEA	59.8	2.2	269.3	6.4
NDIPA	34.7	1.1	208.4	3.0
MNBA	16.0	2.7	96.0	4.3
NPYR	64.5	2.6	193.4	11
NMOR	44.7	1.9	201.2	5.3
NDPA	24.0	2.1	72.1	11
NPIP	50.0	2.9	225.1	6.2
ENBA	10.0	5.3	59.9	3.3
NDBA	59.5	3.5	208.2	4.8

**Table 3:** Intermediate precision of NAs by GC-MS method (n = 10).

NAs	C (µg/L)	RSD (%)
NDMA	48.30	5.9
NMEA	60.00	6.5
NDEA	119.70	8.0
NDIPA	69.50	6.6
MNBA	32.00	8.7
NPYR	128.90	7.1
NMOR	89.40	5.6
NDPA	48.00	6.4
NPIP	100.00	7.7
ENBA	20.00	8.8
NDBA	119.00	8.7

**Table 4:** Recovery of NAs in ultrapure water and drinking water by LLE-GC-MS.

NAs	C (µg/L)	Ultrapure Water (n = 10)		Drinking Water (n = 29)	
		Rec (%)	RSD (%)	Rec (%)	RSD (%)
**NDMA**	48.3	57	7.9	47	18.3
NMEA	60.0	59	7.7	62	9.4
NDEA	119.7	106	14.5	112	14.2
NDIPA	69.5	103	12.9	115	14.5
MNBA	32.0	94	7.8	108	18.7
NPYR	128.9	93	17.0	101	13.5
NMOR	89.4	83	12.5	83	12.8
NDPA	48.0	97	15.0	104	14.1
NPIP	100.0	103	13.4	115	13.9
ENBA	20.0	82	13.0	89	19.2
NDBA	119.0	103	13.4	125	11.6

**Table 5:** The limit of quantification (LOQ) and method quantification limit (MQL) of NAs by LLE-GC-MS.

NAs	LOQ (µg/L)	MQL (µg/L)	MCT_Tap_ (µg/L)
NDMA	0.0060	0.20	0.30
NMEA	0.0092		
NDEA	0.033		
NDIPA	0.0089		
MNBA	0.0083		
NPYR	0.033		
NMOR	0.019		
NDPA	0.012		
NPIP	0.028		
ENBA	0.0045		
NDBA	0.038		

**Table 6:** Material parameters required for migration test calculation.

Material	Surface Area (dm^2^)	N	Volume (dm^2^)	S/V Ratio (dm^−1^)	Fc (dia.dm^−1^)
**A**	18.35	4	4	4.6	1
**B**	22.25	4	4	5.6	1
**C**	19.20	4	4	4.8	0.01
**D**	6.41	4	5	5.1	1

N—number of test pieces used together in a migration. Fc—Conversion factor.

**Table 7:** Concentration of NAs in chlorinated water (CW) and demineralized water (DW) in the migration waters of materials C and D analyzed by LLE-GC-MS.

Material	NAs	$a_{n}^{T}$ (µg/L)					
		1st Migration		2nd Migration		3rd Migration	
		CW	DW	CW	DW	CW	DW
**C**	MNBA	106	97	79	47	50	23
	NMOR	<LOQ	57	<LOQ	<LOQ	<LOQ	<LOQ
	NDPA	76	30	51	<LOQ	<LOQ	<LOQ
	ENBA	267	99	142	73	58	39
**D**	MNBA	<LOQ	21	<LOQ	<LOQ	<LOQ	16
	NDBA	<LOQ	<LOQ	65	<LOQ	<LOQ	<LOQ

**Table 8:** Migration rate of NAs from materials in chlorinated (CW) and demineralized water (DW) of materials C and D analyzed by LLE-GC-MS.

Material	NAs	Migration Rate (µg dm^−2^d^−1^)						C_Tap_	
		1st Migration		2nd Migration		3rd Migration			
		CW	DW	CW	DW	CW	DW	CW	DW
**C**	MNBA	7.1	6.5	4.6	2.7	3.3	1.4	0.030	0.010
	NMOR	<LOQ	3.9	<LOQ	<LOQ	<LOQ	<LOQ	---	---
	NDPA	5.3	1.9	3.3	<LOQ	<LOQ	<LOQ	---	---
	ENBA	18.6	6.9	9.9	5.1	4.0	2.7	0.040	0.030
**D**	MNBA	1.4	<LOQ	<LOQ	<LOQ	<LOQ	<LOQ	---	---
	NDBA	<LOQ	<LOQ	4.2	<LOQ	<LOQ	<LOQ	---	---

**Table 9:** Characteristics of the four organic materials (A, B, C and D) used in the migration tests.

Parameter	Material			
	A	B	C	D
Length (mm)	150	158	150	209
Width (mm)	147	158	150	147
Height/Thickness (mm)	3	0	5	3
Color	Grey	White	Black	Beige
Opacity	Opaque	Glossy	Opaque	Glossy
Characteristics	Pure polyurea-based membrane	100% polyurea-based membrane	Flexible rubber seal	Bi-component polyurethane membrane
Suggested use	Waterproof coating for waterworks, roofs, and bridge decks	Impermeable coating for drinking water tanks	High-strength sealant for applications in sewers and drinking water tanks	Biocomponent for the production of 100% polyurea coatings

**Table 10:** Conditions of NA analysis by GC-MS method.

Nitrosamine	Retention Time (min)	Time Window (min)	Quantitation Ion (*m*/*z*)	Confirmation Ion (*m*/*z*)
**NDMA**	10.13	9.5–12.99	42	74
**NMEA**	13.61	13–16.39	42	88
**NDEA**	16.70	16.40–20.99	42	102
**NDIPA**	21.88	21–23.14	43	70
**MNBA**	23.85	23.15–24.99	77	106
**NPYR**	23.90		41	100
**NMOR**	24.01		56	86
**NDPA**	24.14		43	70
**NPIP**	25.66	25–30.99	55	114
**ENBA**	26.39		106	121
**NDBA**	31.47	31–32.99	57	84

## Data Availability

The data on NAs in migration waters are private due to the identification of materials.
